# Rituximab and dupilumab improve eosinophilic granulomatosis with polyangiitis with multiple pulmonary thrombi

**DOI:** 10.1186/s13223-021-00639-x

**Published:** 2022-02-26

**Authors:** Sei Adachi, Chiyako Oshikata, Takeshi Kaneko, Naomi Tsurikisawa

**Affiliations:** 1 Department of Allergy and Respirology, Hiratsuka City Hospital, 1-19-1 Minamihara, Hiratsuka, Kanagawa 254-0065 Japan; 2 Department of Pulmonology, Yokohama City University Graduate School of Medicine, 3-9 Fukuura, Kanazawa-ku, Yokohama, Kanagawa 236-0004 Japan

**Keywords:** Cyclophosphamide, Dupilumab, Eosinophilic granulomatosis with polyangiitis, Immunoglobulin, Pulmonary thrombosis, Rituximab, Venous thrombus

## Abstract

**Background:**

Eosinophilic granulomatosis with polyangiitis (EGPA) is characterized by a necrotizing vasculitis with tissue and peripheral blood eosinophilia affecting small and medium-sized arteries, capillaries, and veins. Venous thromboembolic events are uncommon in EGPA. Moreover, there are only a few reported cases of EGPA complicated by pulmonary embolism or infarction.

**Case presentation:**

We report the case of a 43-year-old woman with eosinophilic granulomatosis with polyangiitis and acute respiratory and heart failure due to bilateral pulmonary artery thrombosis and left femoral vein thrombosis 12 years after disease onset. She also had cardiac involvement (myocarditis, pericardial effusion, and diastolic dysfunction), gastrointestinal symptoms, and peripheral neuropathy. The condition was refractory to treatment with systemic corticosteroids, intravenous cyclophosphamide, and mepolizumab, but the thrombosis and associated acute cardiac failure, as well as the cardiac and gastrointestinal symptoms and multiple polyneuropathy, improved after a switch to rituximab. However, the heart failure did not improve sufficiently and the patient continued to need inhaled oxygen at 1 L/min and asthma exacerbations occurred. We then swapped the patient’s mepolizumab treatment for dupilumab. Not only did she have no further asthma attacks after switching to dupilumab, but also her vasculitis symptoms improved. Oxygen therapy was discontinued as the heart failure improved 5 months after starting the dupilumab.

**Conclusions:**

This may be the first case report of the successful treatment by rituximab of pulmonary thromboembolism associated with EGPA. In addition, in this patient, treatment with dupilumab was effective not only for the asthma symptoms but also for the symptoms of vasculitis and heart failure.

## Background

Eosinophilic granulomatosis with polyangiitis (EGPA) is characterized by a necrotizing vasculitis with tissue and peripheral blood eosinophilia affecting small and medium-sized arteries, capillaries, and veins [1]. Venous thromboembolic events are relatively uncommon; they have occurred in 19 of 232 (8.2%) patients with EGPA [2]. Moreover, there are only a few reported cases of EGPA complicated by pulmonary embolism or infarction [3–5].

Here, we report the case of a female EGPA patient with acute respiratory and heart failure due to bilateral pulmonary artery thrombosis and left femoral vein thrombosis occurring 12 years after disease onset. The condition was refractory to treatment with systemic corticosteroids, intravenous cyclophosphamide (IVCY), and mepolizumab, but the thrombosis and associated acute cardiac failure improved after a switch to rituximab and dupilumab.

## Case report

A 43-year-old Japanese woman had had bronchial asthma since age 3 years, atopic dermatitis since age 13, and allergic rhinitis since age 1. She experienced asthma exacerbations several times a year and had been treated with only short-acting -stimulants as needed from age 20 onward. She had not had severe exacerbations that required hospitalization or intensive care unit admission. At age 29, the woman had developed a persistent slight fever of 37.8 °C, general malaise, headache, abdominal pain, diarrhea, orbital pain, dyspnea, and chest pain. Two months later, she experienced paralysis and numbness in the right hand and both lower limbs and discomfort in the left fingertips. Laboratory tests at the time of onset of these additional symptoms revealed leukocytosis (6790/µL, of which 20.2% were eosinophils) and a positive myeloperoxidase-anti-neutrophil cytoplasmic antibody (MPO-ANCA) test (21 U/mL, normal: 3.5 IU/mL or less). The serum total IgE level was 91.4 IU/mL (normal: 173 IU/mL or less). Antigen-specific serum IgE to mites was detected at 32.70 UA/mL. Chest computed tomography (CT) showed no abnormalities in the lung field, but transbronchial lung biopsy showed interstitial pneumonia with eosinophil infiltration, and bronchoalveolar lavage yielded 27.7% eosinophils. On disease diagnosis a small rash was found only on the left thigh; it was not palpable. Skin biopsy showed perivascular eosinophil infiltration. Upper and lower gastrointestinal endoscopy revealed dark red signs in the stomach and duodenum, but no endoscopic abnormalities were present in the lower gastrointestinal tract. Biopsy revealed submucosal infiltration of eosinophils in the stomach, duodenum, appendix, and descending colon. Echocardiography showed an ejection fraction of 56.5%, pericardial effusion, pericardial thickening, and diastolic dysfunction. Cardiac scintigraphy with iodine-123-labeled MIBG (metaiodobenzylguanidine) revealed cardiac involvement, appearing as spotty accumulation of MIBG in the anterior wall region and the interventricular septum. The patient was diagnosed according to the Japanese diagnostic criteria for AGA/CSS (allergic granulomatous angiitis/Churg-Strauss syndrome) as having EGPA [6]. The patient had normal lung function at diagnosis when not experiencing asthma attacks, including a VC (vital capacity) of 3.07 L; %VC of 111.6%; FEV_1_ (forced expiratory volume in 1 s) of 2.87 L; %FEV_1_ of 99.9%; V_50_ (maximum expiratory flow rate at 50% of forced vital capacity) of 3.75 L; and %V50 of 93.7%. She underwent bronchial provocation testing with acetylcholine and histamine. Bronchial sensitivity was expressed as the PC_20_, defined as the provocative concentration of agonist in the inhaled aerosol (acetylcholine at up to 20 mg/mL or histamine at up to 10 mg/mL) leading to a 20% decrease in FEV_1_. The results were positive: PC_20_-acetylcholine was 18.907 mg/mL and PC_20_-histamine was 1.806 mg/mL.

**Fig. 1:**
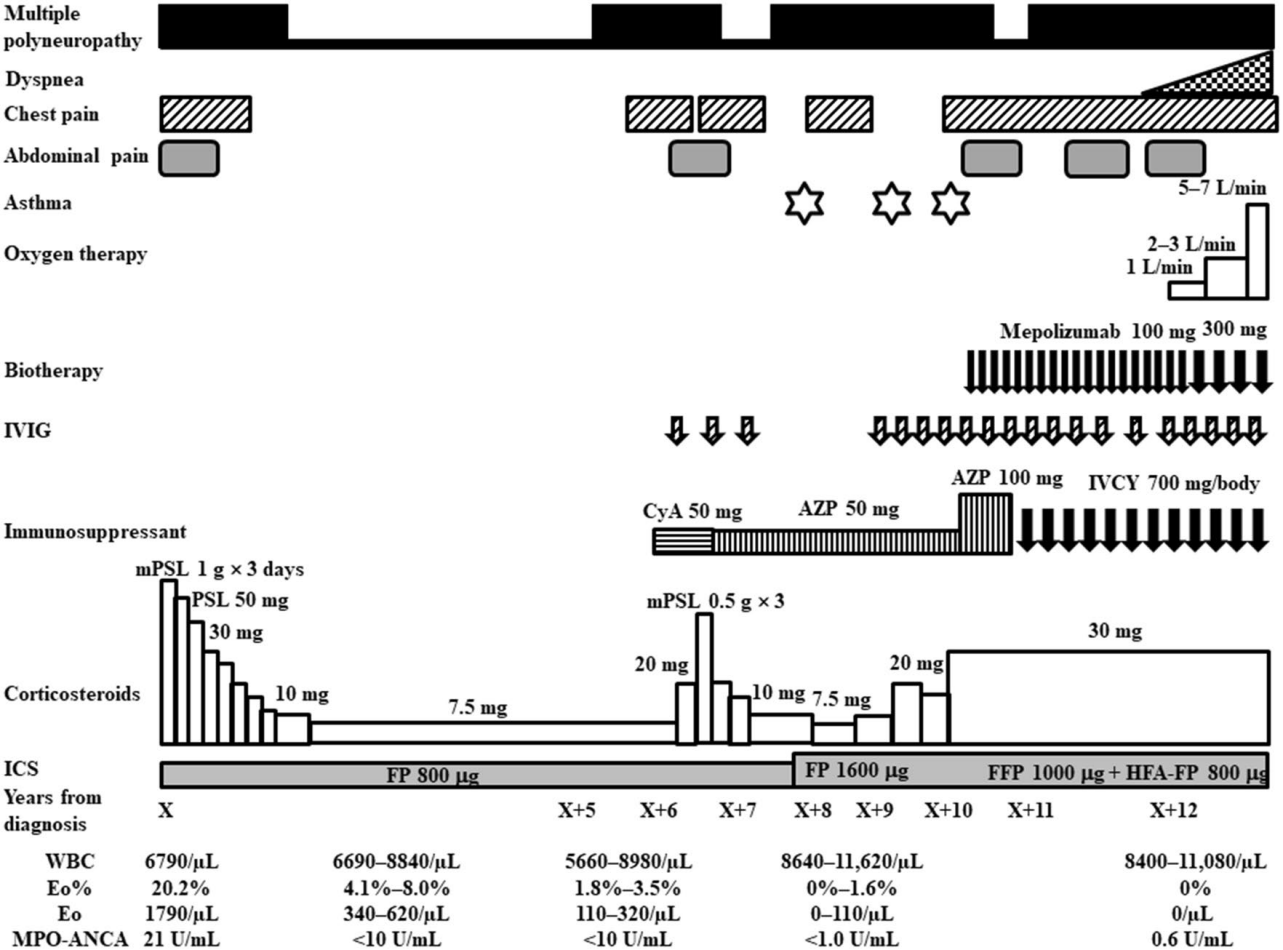
Clinical course of the patient from the time of diagnosis of eosinophilic granulomatosis with polyangiitis to the time of heart failure. *AZP* azathioprine, *CyA* cyclosporine A, *Eo* eosinophil, *FFP* formoterol fluticasone combination, *FP* fluticasone propionate, *HFA* hydrofluoroalkane, *ICS* inhaled corticosteroid, *IVCY* intravenous cyclophosphamide, *IVIG* intravenous immunoglobulin, *MPO-ANCA* myeloperoxidase-anti-neutrophil cytoplasmic antibody, *mPSL* methylprednisolone, *PSL* prednisolone, *WBC* white blood cell count

**Fig. 2:**
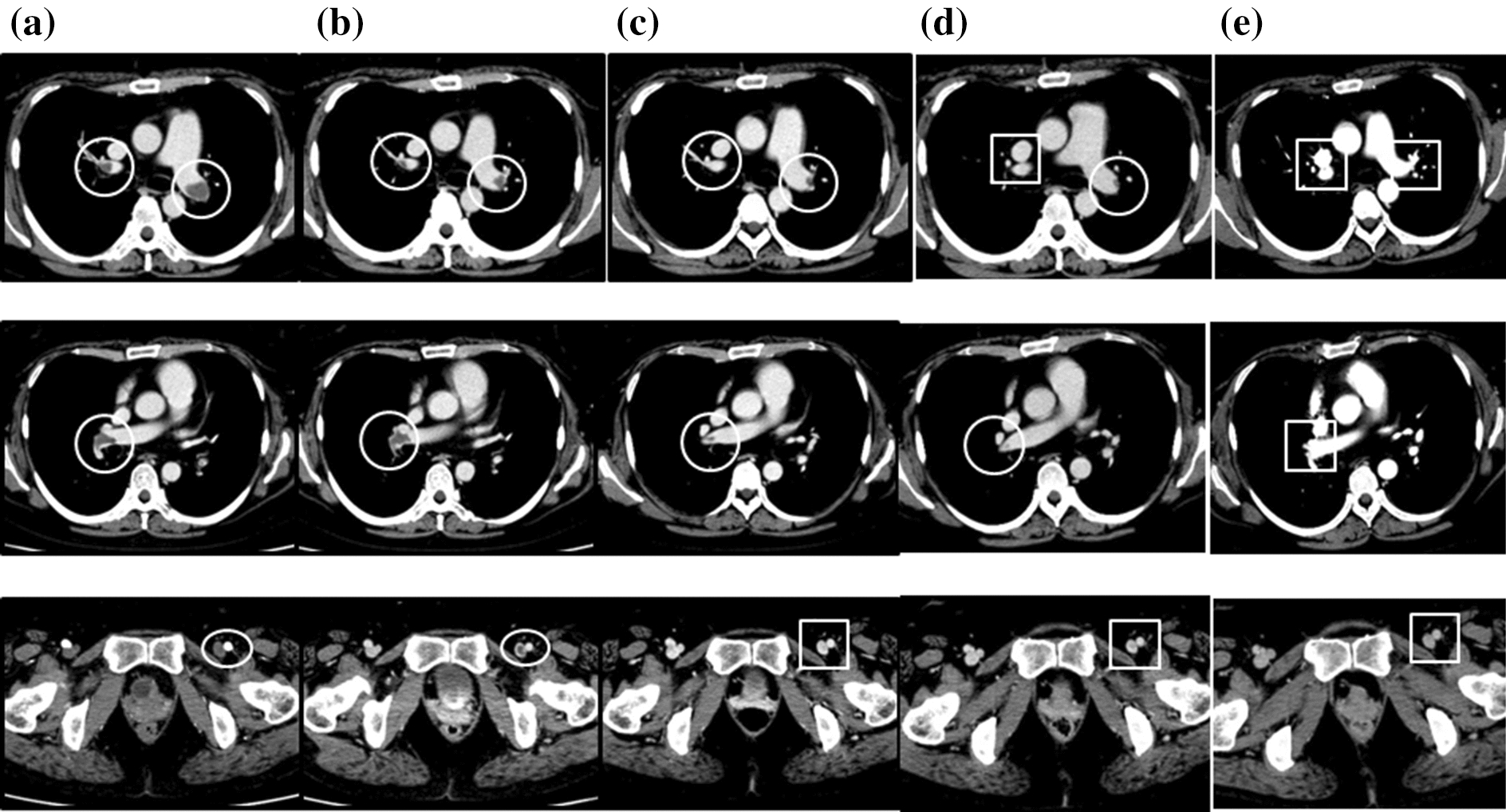
Rituximab-associated changes in the thrombi in both pulmonary arteries and the left common femoral vein thrombus on contrast-enhanced computer tomography (CT). Contrast-enhanced CT images before rituximab initiation (**a**), immediately after the first course of rituximab (500 mg/week for 4 weeks, repeated 6-monthly) (**b**), and at 3 months (**c**), 1 year (**d**), and 2 years 2 months (**e**) after the start of rituximab. The thrombi observed before rituximab initiation immediately started to improve with rituximab therapy. Open circles, thrombus present; open squares, thrombus has disappeared

**Fig. 3:**
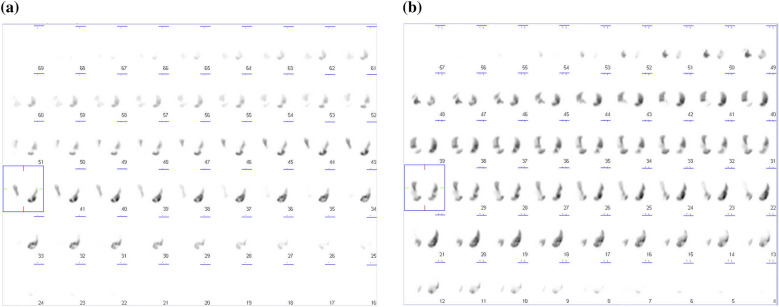
Lung perfusion scintigraphy before and after rituximab initiation. There were multiple regions of decreased blood flow in both lungs, but predominantly on the right side, before rituximab administration (**a**). Two years and 2 months after rituximab initiation, blood flow had improved but some areas of decreased flow remained (**b**)

**Fig. 4:**
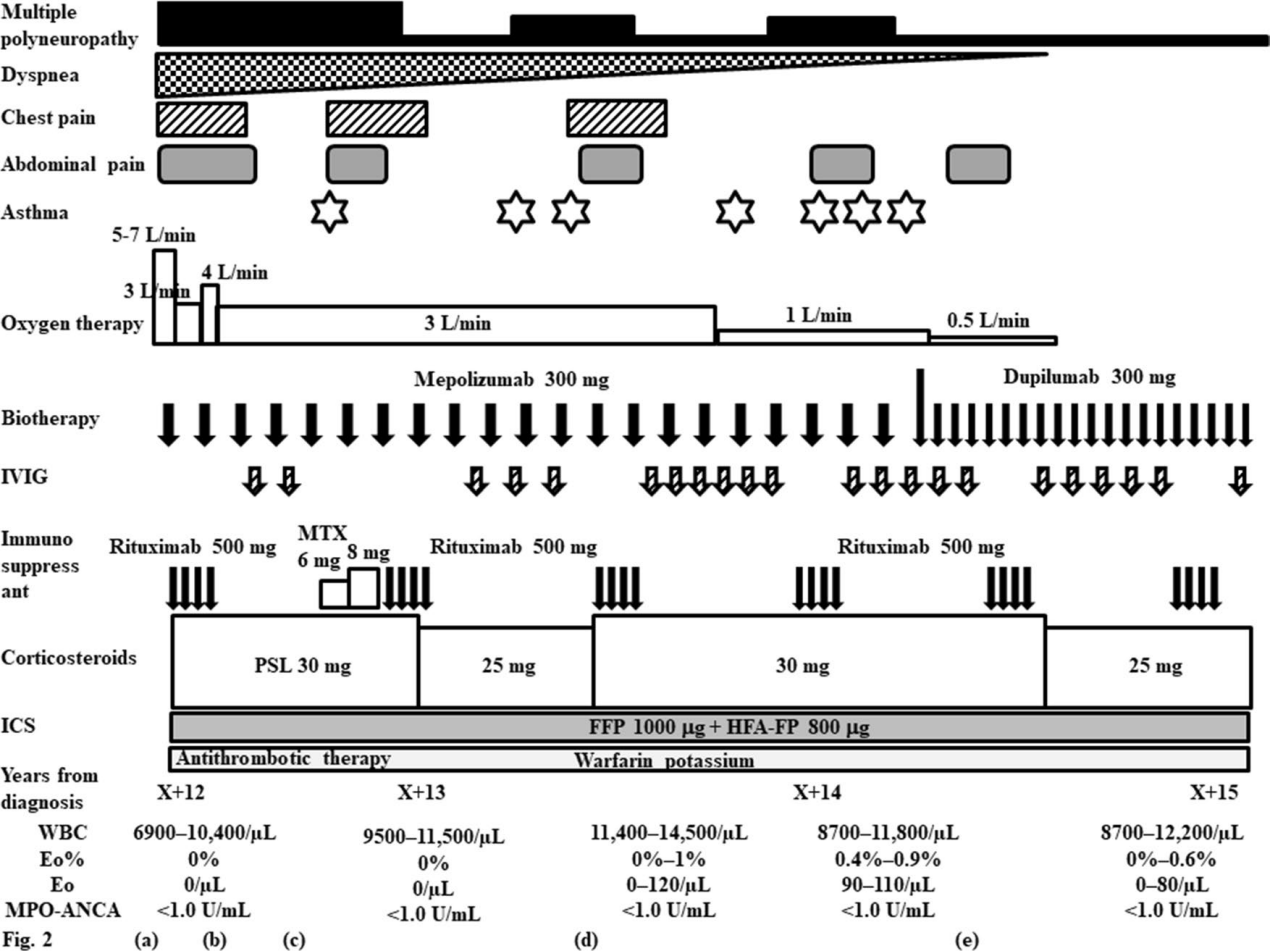
Clinical course of the patient from the time of development of thrombi in both pulmonary arteries to the time of last examination. *Eo* eosinophil, *FFP* formoterol fluticasone combination, *FP* fluticasone propionate, *HFA* hydrofluoroalkane, *ICS* inhaled corticosteroid, *IVIG* intravenous immunoglobulin, *MPO-ANCA* myeloperoxidase-anti-neutrophil cytoplasmic antibody, *MTX* methotrexate, *PSL* prednisolone, *WBC* white blood cell count

After treatment with pulsed methylprednisolone (1000 mg intravenously daily for 3 consecutive days), followed by 50 mg/day orally of prednisolone and daily treatment with 800 μg inhaled fluticasone propionate without long-acting beta agonist or leukotriene receptor antagonist for asthma, the patient’s mononeuritis multiplex, as represented by paresthesia in both lower limbs and numbness in the right hand, and the cardiac involvement, as indicated by general malaise, chest pain, and dyspnea, gradually improved. Because the patient planned to become pregnant, we initially did not treat her with additional immunosuppressants such as IVCY. She remained in remission for 5 years on prednisolone 7.5 mg, with an eosinophil count ranging from 340 to 620/µL (leukocyte count 6690 to 8840/µL). However, 6 years after diagnosis the patient relapsed, with chest pain, abdominal pain, and paralysis and numbness in the right hand and numbness in both lower limbs. The eosinophil count was 320µL (leukocyte count 8980/µL, of which 3.5% were eosinophils). We were unable to continue administration of 50 mg cyclosporine due to side effects of headache. We increased the daily prednisolone dose to 20 mg and added 50 mg of azathioprine increasing to 100 mg, as well as several cycles of intravenous immunoglobulin (IVIG) (400 mg/kg for 5 consecutive days) at a 3- or 4-month interval, but the woman’s vasculitis, heart and gastrointestinal symptoms, and peripheral neuropathy did not improve, and she sometimes had asthma exacerbations. Eight years after diagnosis we increased the daily dose of inhaled corticosteroid to 1600 mg of fluticasone propionate. We defined asthma exacerbation as the presence of paroxysmal dyspnea and wheezing. Ten years after diagnosis, the patient’s daily dose of prednisolone was increased to 30 mg, the azathioprine treatment was switched to IVCY (600 mg/m^2^) every 3 to 4 weeks, and once monthly IVIG at the abovementioned dose and monthly mepolizumab at 100 mg increasing to 300 mg were added. However, the vasculitis symptoms did not improve and the patient developed heart failure, requiring oxygen at 5–7 L/min (Fig. 1). Twelve years after diagnosis we confirmed the presence of thrombi in both pulmonary arteries and the left common femoral vein on contrast-enhanced CT (Fig. 2a), as well as multiple regional decreased blood flow in both lungs, predominantly on the right side, on lung perfusion scintigraphy (Fig. 3a). The number of eosinophils did not increase (leukocyte count 11,080/µL, of which 0% were eosinophils). The patient did not have any risk factors for thrombosis, and we examined protein C (50%, normal: 64–146%), protein S (121%, normal: 60–150%), lupus anticoagulant (1.0, normal: < 1.2), anti-beta2 glycoprotein (< 1.2, normal: < 3.5) or anti-cardiolipin antibodies (< 1.2, normal: < 3.5) in the serum. We were unable to test for mutation in the prothrombin gene and factor V Leiden. The patient’s IVCY was replaced with rituximab 500 mg once a week for 4 weeks 12 years after diagnosis (Fig. 4), in addition to 3 mg/day of warfarin potassium and continued treatment with prednisolone 30 mg/day and IVIG every 1–2 months. Mepolizumab 300 mg/month was added 10 years after diagnosis. The heart failure began to improve 2 weeks after the initiation of rituximab the patient’s oxygen demand had decreased from 7 L/min to 3 L/min (Fig. 4). However, her symptoms of cardiac involvement and peripheral neuropathy and her gastrointestinal symptoms remained. She received 6 courses of rituximab (once weekly for 4 weeks every 6 months), but her heart failure did not improve and she continued to need inhaled oxygen at 1 L/min 14 years after diagnosis; asthma exacerbations occurred frequently despite the administration of mepolizumab, and there was no overall improvement in the vasculitis symptoms. At 14 years after diagnosis the number eosinophils had not increased (leukocyte count 8700 to 11,800/µL, of which 0.4% to 0.9% were eosinophils). The patient had obstructive impairment of lung function (%FEV_1_, 76.2%) and high level of 40 ppb in fractional exhaled NO, which we had not measured at the time of diagnosis. The serum total IgE level was greatly reduced at < 5 IU/mL, and no antigen-specific serum IgE to mite allergen was detected.

Fourteen years after diagnosis we changed the patient’s treatment with mepolizumab 300 mg/month to dupilumab 300 mg every 2 weeks to treat the asthma exacerbation. Not only did she have no asthma attacks immediately after switching to dupilumab, but also her vasculitis symptoms, including residual abdominal pain, dyspnea on effort, and leg edema, numbness, and paralysis, improved. The patient received additional treatment with methotrexate for only two months after the first course of rituximab for relapse of heart failure.

Oxygen therapy was discontinued, as by 5 months after the patient had started the dupilumab her heart failure had improved. She was able to reduce her daily dose of prednisolone to 25 mg (Fig. 4). The number of eosinophils did not increase (leukocyte count 8700 to 11,800/µL, of which 0.4% to 0.9% were eosinophils) 1–6 months after the start of the dupilumab. The serum total IgE level did not change after administration of dupilumab. One month after the start of the dupilumab, the %FEV_1_ had improved to 85.9% and the fractional exhaled NO had decreased to 23 ppb.

The thrombi in both pulmonary arteries and the left common femoral vein had improved by 1 month after rituximab initiation (Fig. 2b), and the thrombus in the left common femoral vein had disappeared by 3 months after rituximab initiation (Fig. 2c). The thrombus in the right pulmonary artery had disappeared by 1 year after rituximab initiation (Fig. 2d), and the thrombus in the left pulmonary artery had disappeared by 2 years and 2 months after the start of rituximab (4 months after the change from mepolizumab to dupilumab) (Fig. 2e). However, some multiple regional decreased blood flow on lung perfusion scintigraphy remained 2 years and 2 months after rituximab initiation (Fig. 3b).

## Discussion and conclusions

It has been reported that 65.6% of venous thrombi in ANCA-related vasculitis occur within 1 year of diagnosis [7]. Arterial and venous thrombosis in EGPA can occur up to 2 years before or after disease diagnosis [8]. To our knowledge, there have been no previous reports of severe pulmonary thrombosis 12 years after a diagnosis of EGPA. We did not treat the patient with immunosuppressants (e.g. IVCY) initially, because at the time of diagnosis she intended to become pregnant, and her vasculitis symptoms were stable for more than 6 years with steroid treatment without immunosuppressants. However, after 6 years, although we still withheld the IVCY, we considered it necessary to give azathioprine or methotrexate [9]. Thrombosis is rarely an adverse effect of immunoglobulin administration [10]. We considered that IVIG administration might strip immune complex from the vascular endothelium, as it has been reported to neutralize autoantibodies [11] and reduce the abundance of immune complexes [12] in the blood.

Although there was a report that glucocorticoids might be associated with an increased incidence of venous thromboembolism, available data did not allow this hypothesis [13]. Epidemiologic data on increased risk of venous thromboembolism in patients with systemic vasculitis have accumulated in recent years. However, the action of immunosuppressive agents in modulating the risk of **v**enous thromboembolism in patients with systemic vasculitis is not yet clear except for Behçet’s disease [14].

Most patients with eosinophilic pneumonia have radiological findings in the lungs, but in some of these people chest X-rays or CT scans can remain normal throughout the course of the disease [15]. Therefore, if possible, we also perform lung biopsy and bronchoalveolar lavage even if there are no findings in the lung field on CT.

This patient had neither an increase in the eosinophil count at relapse nor any risk factors for thrombosis other than vasculitis from the time before the onset of vasculitis to the time of all relapses. Eosinophilia in the peripheral blood is not included in the definition of relapse of vasculitis [16], and we confirmed that the number of eosinophils does not necessarily increase when EGPA relapses [17]. The association between the MPO-ANCA titer and disease severity has not been well evaluated [18].

We confirmed every 4 weeks that the patient’s heart failure symptoms gradually improved from the time of rituximab initiation, and we considered that the symptoms of heart failure were probably improved by the rituximab rather than by the warfarin. It is true there are not guidelines or clear recommendations on the treatment of thrombotic events in eosinophilic disorders by Réau et al. [19] reported that, in eosinophil-related disease, treatment of venous thrombosis, including pulmonary embolism, with vitamin K antagonists (72.5% of patients) was more common than treatment with oral non-vitamin K antagonists (20.0%) or low-molecular-weight heparin (7.5%). In accordance with this report, we gave warfarin to our patient, not low-molecular-weight heparin.

This case of EGPA complicated by pulmonary thrombosis was treated with steroids, IVCY, mepolizumab, IVIG, and methotrexate, but we confirmed that the patient’s symptoms of heart failure improved after we switched her from IVCY to rituximab. Patients with EGPA complicated by pulmonary embolism have been treated with systemic corticosteroids or IVCY, or both [3–5], and rituximab may induce remission in patients with relapsing or refractory EGPA [20]. One study found that ANCA-positive EGPA patients are more likely than ANCA-negative EGPA patients to achieve remission by treatment with rituximab [21]. However, in only one study there has been no clear significant difference in remission rates between ANCA-positive and -negative patients [20]. Some of patients with refractory or relapse ANCA-negative EGPA achieved remission in a systemic search of the English literature [22].

In this case, it might be possible that the MPO-ANCA titer was low because the diagnosis was made very early. Although the MPO-ANCA titer in our patient was low positive from the time of diagnosis, treatment with rituximab was effective for pulmonary thrombosis. We considered that in this patient there was no relationship between ANCA positivity and the effect of rituximab in achieving remission. We reported that the percentages of activated B cells increased in patients with frequently relapsing EGPA both MPO-ANCA-negative and -negative [23]. We considered that rituximab might contribute to activated B cell reconstruction. We showed that increased peripheral blood ILC2 count was associated with disease activity in EGPA [17]. Peripheral blood ILC2 numbers in asthma patients with treatment of dupilumab was lower than in those without treatment of dupilumab [24]. We considered that administration of dupilumab reduces ILC2 and suppresses type 2 inflammation as one of the mechanisms of dupilumab.

To our knowledge, there have been no previous reports of the effectiveness of rituximab in EGPA patents with pulmonary thromboembolism. Moreover, we have found only one report of success with dupilumab for EGPA, in two pediatric cases (a 13-year-old boy and a 16-year-old girl) [25]. Here, we encountered a rare case of pulmonary thromboembolism 12 years after the onset of EGPA. This may be the first case report of the successful treatment of EGPA-associated pulmonary thromboembolism by rituximab. In addition, in this patient, treatment with dupilumab was effective not only for the asthma symptoms but also for the symptoms of vasculitis. Further RCTs are needed to verify whether Dupilumab is effective against vasculitis.

Dupilumab can cause a transient increase in the blood eosinophil count [26]. However, our patient had no increase in eosinophil numbers in the peripheral blood and had no side effects from dupilumab initiation. This may have been because of her concurrent high dosage of prednisolone. This study did not report health hazards associated with dupilumab initiation [26].

## Data Availability

Not applicable.

## Abbreviations

CT: Computed tomography
EGPA: Eosinophilic granulomatosis with polyangiitis
IVCY: Intravenous cyclophosphamide
IVIG: Intravenous immunoglobulin
MPO-ANCA: Myeloperoxidase-anti-neutrophil cytoplasmic antibody
